# Practice variation in long‐term care access and use: *The role of the ability to pay*

**DOI:** 10.1002/hec.3940

**Published:** 2019-08-30

**Authors:** Daisy Duell, Maarten Lindeboom, Xander Koolman, France Portrait

**Affiliations:** ^1^ Department of Life Sciences Vrije Universiteit Amsterdam (VU), Talma Institute Amsterdam the Netherlands; ^2^ Department of Economics Vrije Universiteit Amsterdam (VU) Amsterdam the Netherlands; ^3^ Tinbergen Institute Amsterdam the Netherlands; ^4^ Centre for Health Economics Monash University Victoria Australia

**Keywords:** health care, health care financing, inequality, regional consumer behaviour

## Abstract

Practice variation in publicly financed long‐term care (LTC) may be inefficient and inequitable, similarly to practice variation in the health care sector. Although most OECD countries spend an increasing share of their gross domestic product on LTC, it has received comparatively little attention to date compared with the health care sector. This paper contributes to the literature by assessing and comparing regional practice variation in both access to and use of institutional LTC and investigating its relation with income and out‐of‐pocket payment. For this, we have access to unique individual‐level data covering the entire Dutch population. Even though we found practice variation in the use of LTC once access was granted, the variation between regions was still relatively small compared with international standards. In addition, we showed how a co‐payment measure could be used to reduce practice variation across care office regions and income classes making the LTC system not only more efficient but also more equitable.

## 1 INTRODUCTION

Practice variation in health care refers to variation across geographic areas, or between doctors, that cannot be explained by differences in need (Skinner, 2011). As a result, some regions might use less care than expected given their populations' need for care, and other regions might be using more care than would be expected given their populations' needs. Even though practice variation is, to date, predominantly studied in health care, the same dynamics apply to long‐term care, which is the focus of this paper.

Practice variation is an issue for policymakers if it leads to an inefficient or inequitable allocation of health care, for instance, if it systematically relates to (illegitimate) causes such as income or ethnicity (Braveman & Gruskin, 2003). In such circumstances, policy makers should be concerned because resources are not being used where they are most needed. If they can address the problem effectively then a redistribution of resources may increase allocative efficiency. By limiting practice variation, especially if it systematically relates to these unwarranted causes, it is, ceteris paribus, likely to result in a higher utility and a more equitable distribution of resources.

Numerous empirical studies have shown substantial levels of practice variation in the health care sector (see for instance the systematic review in Corallo et al., 2014; Wennberg, 2010; Johnson & Stukel, 2016). All these contributions look at different aspects of the health care sector, such as variation in screening and diagnostic testing, hospital admissions, and surgical procedures. Much less evidence is available for practice variation in various long‐term care (LTC) settings, even though it is equally concerning for policy makers.

The few available studies on LTC mostly focused on the use of (or expenditures on) LTC. Jensen and Lolle (2013), Trydegård and Thorslund (2001), and Davey, Johansson, Malmberg, and Sundström (2006) investigated practice variation in the use of LTC in Denmark and Sweden and showed substantial variations across municipalities. Jensen and Lolle (2013) showed that twice as much was spent on elderly care in the Danish municipality with the highest spending compared with the municipality with the lowest while controlling for population characteristics. They could only explain half of the variation. Further, a substantial share of the practice variation was associated with the municipality's wealth. In a previous study of the Netherlands, Duell, Koolman, and Portrait (2017) explored practice variation in granted entitlements for (i.e., access to) Dutch institutional LTC. The authors found comparatively small levels of practice variation (up to 3%) across care office regions compared with international standards and that only a minor part of the limited practice variation in access could be accounted for by regional supply differences in care facilities. The current paper adds to this research by looking at practice variation not only in the entitlements granted but also in the entitlements actually used.

As in most OECD countries, the majority of the Dutch LTC spending is on institutional LTC ([dataset] OECD.stat, 2017). The Netherlands differs from most other countries in that a comparatively high share of the institutional LTC expenditure is funded publicly ([dataset] OECD.stat, 2017). In this paper, we are seeking for opportunities to improve the efficiency and the equity of the Dutch LTC system. In order to do that, we would like to present evidence for practice variation from the Dutch LTC. The Dutch system provides an interesting case study because the way it is structured allows us to separately analyse (a) the more centralized and standardized needs‐assessment procedure (which grants access) and (b) a regional provision of LTC (which regulates use). Both parts of the system involve different stakeholders and different incentives, which may result in differences in practice variation. The Dutch system has a national independent agency that uses a standardized needs‐assessment procedure to grant *access* to LTC (Colombo, Llena‐Nozal, Mercier, & Tjadens, 2011; Dutch Government, 2005). This makes it well‐suited to limit regional practice variation (Bakx, Douven, & Schut, 2016). However, the process of organizing the use of LTC is more decentralized, organized by over 30 regional care offices. The regional budgets are “open‐ended” and hence the institutional LTC granted should legally be accessible to every client. However, the actual use may also be dependent on differences in preferences and variation in the local provision of LTC.

The first main contribution of our paper is to assess and compare practice variation in both access to and use of institutional LTC across Dutch care office regions, given that the client has applied for LTC. We hypothesize that practice variation is larger in used than in granted Dutch institutional LTC (our first hypothesis). The differences in systems for allocating entitlements (a nationally regulated process) and for actually using care (a more locally organized process) make the Netherlands an interesting country to study.

Many studies have shown that individuals with lower income are much more likely to use care than individuals with higher incomes (for instance: van Doorslaer et al., 2006; Portrait, Lindeboom, & Deeg, 2000; Schoen et al., 2010). As the ability to pay is often not evenly distributed across regions ([dataset] CBS, 2014), this may explain part of the practice variation in LTC use. The second main contribution of our paper is therefore to investigate whether and how the client's financial situation (characterized by income and the amount of out‐of‐pocket payments that a client has to pay) is associated with practice variation in LTC use in the Netherlands. We hypothesize that individuals with a better financial situation comparatively use less formal reimbursed care, because they may be able to arrange private care (our second hypothesis; van Doorslaer et al., 2006). In addition, we hypothesize that out‐of‐pocket payments reduce the practice variation in use of institutional LTC across care office regions (our third hypothesis). Understanding whether and how practice variation is associated with differences in financial situations is important, as it may help to develop policies aimed at reducing unequitable access to LTC.

We have access to uniquely detailed administrative datasets covering the entire Dutch population. Two of these datasets include information on all granted institutional care (IC) in 2013 and on the amount and type of granted care actually used, given that the client has applied for LTC. Furthermore, these datasets provide a rich set of demographic, socio‐economic, and health characteristics on all included individuals, which allows us to comprehensively correct for differences in needs between care office regions and are essential to accurately determine practice variation, which could lead to allocative inefficiency. The need assessment is performed by highly qualified professional assessors and the datasets allow us not only to have access to the information on which they base their assessment but also to the results of their assessments. A third dataset provides detailed individual information on the client's financial situation, including income and out‐of‐pocket payments.

In summary, we (a) provide separate insight into practice variation for granted and for used institutional LTC entitlements across Dutch care office regions, given that the client has applied for LTC and (b) investigate whether differences in income and in out‐of‐pocket payments can explain practice variation. The study results will contribute to the literature by revealing differences in practice variation between access to LTC, which is centrally regulated and managed, and LTC use that is affected by other factors like patient preferences. This difference in systems, combined with the explanatory power of out‐of‐pocket payments and income regarding practice variation, results in more insight into the efficiency and equitability of access to and use of institutional LTC and a better understanding of the ways to control practice variation in LTC.

The following section provides a background to the Dutch LTC system and policy measures taken to control the increasing financial burden. In Section 3, we discuss the data and methodology that we exploited to provide insight into practice variation for granted and used institutional LTC and the influence of income and in out‐of‐pocket payments. Section 4 shows the results on the basis of these analyses. Finally, Section 5 discusses the results by comparing them with results and conclusions from current scientific literature. In addition, Section 5 also contains our main conclusions.

## 2 BACKGROUND: THE DUTCH LTC SYSTEM AND POLICY MEASURES TO CONTROL LTC COSTS

### 2.1 Granting process

The Dutch LTC system has a national independent agency that uses nationwide criteria and a standardized procedure to grant access to LTC (Colombo et al., 2011; Dutch Government, 2005). Since 2006, the needs assessment for IC has been carried out by an independent assessor from the Care Needs Assessment Centre (CNAC; in Dutch: Centrum Indicatiestelling Zorg). The CNAC grants the entitlements and defines the type and amount of long‐term IC that patients are entitled to. This is a centrally regulated process, where care is granted on the basis of the most important dominant and secondary health problems (Appendix S1), to enable equitable access to LTC across the Netherlands. “Customary care” (or informal care), which includes usual care that partners, parents, adult children, and other household members were expected to provide, was also taken into account in the needs assessments (CNAC, 2013). After the granting process has been completed, regional care offices have to ensure that patients domiciled in their region are able to use the granted institutional LTC (Mot, 2010). These care offices receive a budget that is based on production agreements made in previous years, and additional funding is given when needed in order to enable that clients receive the granted institutional LTC within an acceptable period (Duell et al., 2017; Dutch Health Care Authority, 2013). In addition, if a client needs more, or a different type of, institutional LTC than assessed, a new assessment is carried out centrally (CNAC, 2013). This LTC system's wide coverage of services, combined with an ageing population, has led to an increasing financial burden. In 2017, the OECD reported an increase of 229% (from 0.8% to 2.6% of the GDP) over a period of 15 years (from 2000 to 2015, respectively) in the Netherlands.

### 2.2 Dutch cost sharing initiative

Policy makers can take a number of measures to help control this increasing financial burden. They can for instance strengthen the eligibility criteria (i.e., allowing only people with high need for LTC) or may increase cost sharing initiatives in which insured individuals (clients) pay part of the costs of the received care (Colombo et al., 2011). Until 2013, in the Netherlands, a client had to pay a relatively small share of the total costs out‐of‐pocket (income‐based co‐payments 1In 2013, there were two levels of personal contributions for care provided in an institution and these are defined by the length of stay (on the basis of information from the Decision on Care Contribution Art. 6 and the current calculation of CAK). During the first 6 months of a stay in an institution, the client is required to pay the lower level of out‐of‐pocket payments. In 2013, this “lower rate” personal contribution was a minimum of €152 per month and a maximum of €797.80 per month and was based solely on client and partner income. After the first 6 months of any stay, the client is likely to be required to pay the higher rate of out‐of‐pocket payments, which are not calculated solely on the basis of the clients' income. In 2013, the maximum amount of personal contribution per month was €2,189.20. These out‐of‐pocket payments are calculated on the basis of the income of the client and his/her partner in 2011. In addition to the resulting amount, 8% of a households' capital is added. The first €21,000.00 per person is tax‐free capital and is not included for the purpose of this calculation. The value of a client's home is included in the calculation. Finally, the resulting amount is divided by 12 to yield the amount of out‐of‐pocket payments per month with the maximum of €2,189.20.) in order to receive LTC (Schut, Sorbe, & Høj, 2013). However, to realize savings of €200 million, the Dutch government then decided to base co‐payments not only on income but also on the client's capital. This resulted in a large increase of out‐of‐pocket payments for institutional LTC for some groups of clients (Dutch Government, 2012).

This significant increase in out‐of‐pocket payments addresses the problem of clients consuming LTC, for which the governmental costs exceed the average consumers' marginal value, because the consumer is not the payer. In other words, by increasing the out‐of‐pocket payments, one can spend less on other goods when consuming the same amount of LTC (diminishing marginal utility). Consequently, one only wants to consume the LTC needed in order to maximize the amount one can spend on other goods, increasing ones' marginal utility. In that case, an efficiency gain can be overserved within the LTC system. Note also that Dutch out‐of‐pocket payments for LTC are highly progressive and very low for those with the lowest incomes. These payments are therefore likely to make the LTC system even more equitable.

## 3 DATA AND METHOD

### 3.1 Datasets and study sample

We exploit three unique nationwide datasets. First, the CNAC dataset contains information on all applications for LTC (*n* = 1,582,339) and, more specifically, on all institutional LTC entitlements granted and valid in 2013 in the Netherlands (*n* = 553,819). Second, the Vektis dataset provides information on the amount of insured institutional LTC used/reimbursed in 2013 per individual (*n* = 435,149 valid entitlements in 2013). Third, the CAK data provides data on income and out‐of‐pocket payments at an individual level (*n* = 495,496 when selecting the valid entitlements in 2013). A unique identifier in each dataset allows us to merge the datasets at the level of the entitlement. As a result, we know who was granted a specific LTC entitlement, conditional on the fact that the client has applied for it, the type of the entitlement, and to which extent the individual used it. In addition, the Vektis and CNAC datasets provide detailed information on demographic, health, and social characteristics of applicants. After merging these datasets, our final study sample includes 553,819 entitlements made by 426,934 unique clients. Furthermore, the dataset provides information covering 393 unique municipalities and 32 unique care offices.

Finally, by means of day tariffs, we quantify (in euros) the amount of institutional LTC granted per client in 2013 and compare this to the amount of institutional LTC that was reimbursed (on the basis of the same tariffs). The NZa provides data on day tariffs for care entitlements ([dataset] Dutch Health Care Authority, 2013). Finally, note that both the granted and used amount of care are corrected for the date of death if applicable.

### 3.2 Dependent variables

The first dependent variable ( $y_{j}^{1}$) was the intensity of IC entitlements granted expressed in euro per year and was computed using (a) the day tariff of the entitlement/care granted, times (b) the duration in days of the care granted, times (c) the amount of care granted per week. The second dependent variable ( $y_{j}^{2}$) was the amount of IC used in 2013 in euros. Both of these variables are conditional on the fact that the client has applied for LTC.

### 3.3 Financial characteristics

The variable income (*E*_*j*_) was categorized as the following: (a) unknown, (b) low (<€15,000 per year), (c) under average (€15,000–€30,000 per year), (d) average (€30,000–€45,000 per year), and (e) high (>€45,000 per year); Group 4 was used as the reference category. In addition, the variable “amount of out‐of‐pocket cost due per individual” was also used as an independent variable (*P*_*j*_).

### 3.4 Other independent variables

Factors determining the amount of IC entitlements granted and used should, for the largest part, be related to the needs of a client. The following factors were thus included as case‐mix variables in the analyses:

1. Gender (the value “0” for males and the value “1” for females).
2. Health problem (dominant and secondary) at the time of application was categorized as follows: (a) psychogeriatric problems, (b) psychiatric, (c) physical disability, (d) intellectual disability, (e) sensory disability, (f) psychosocial, and (g) no dominant/secondary health problem registered. Somatic illness was used as a reference group.
3. The following categories were used to characterize marital status: (a) marital status unknown, (b) not married and not registered as partners at this moment, (c) widow, and (d) other. The category married was used as a reference group.
4. Age was divided into 11 categories, each of a 10‐year time span (0–9, 10–19, 20–29, 30–39, 40–49, 50–59, 60–69, 70–79, 90–99, and ≥100; the category 80–89 is used as reference group).

In addition, age, marital status, and gender were included in the analyses as interaction variables. An extra independent variable was also included, taking the value “1” if the entitlement intensity used deviates from the entitlement intensity granted, and taking the value “0” otherwise. This variable indicates whether the type of care actually used deviates from the one the client has been entitled to, and was included in the analyses to control for the fact that a different type of care is used than the one originally granted. This only happens in exceptional circumstances, for example, due to an abrupt change in need, and was therefore included as an additional proxy for health (namely in only 660 observations out of 553,819 observations). In addition, as formal home care may be a likely substitute for institutional LTC, we included an independent variable in the analyses on use. This variable was taking the value “1” if a formal home care entitlement was provided at the time of the needs assessment for institutional LTC, and taking the value “0” otherwise. Finally, month dummies for timing of entitlements were included in the analyses to account for period effects, taking the value “1” if an entitlement is valid in a certain month and taking the value “0” otherwise.

### 3.5 Statistical analyses

#### 3.5.1 Descriptive statistics

We computed descriptive statistics of clients who were granted and who used institutional LTC. In addition, ANOVA was used to check for differences in variables across care office regions. Furthermore, financial characteristics were aggregated by care office region and income classes. To get a first understanding of the data, we performed linear regression analyses between OOP payments and institutional LTC use stratified by income group.

#### 3.5.2 Assessing practice variation

In our main analyses, we computed the average variation in IC granted and IC used per care office region that could not be explained by the differences in health characteristics. This was carried out using linear regression analyses. The regression analyses can be summarized by means of the following equations (1‐2):

(1) $$y_{j}^{1}=\beta_{0}^{1}+\sum_{i=1}^{I}X_{\mathrm{ij}}\beta_{i}^{1}+\sum_{k=1}^{K}M_{\mathrm{kj}}\gamma_{k}^{1}+u_{j}^{1},$$

(2) $$y_{j}^{2}=\beta_{0}^{2}+\sum_{i=1}^{I}X_{\mathrm{ij}}\beta_{i}^{2}+\sum_{k=1}^{K}M_{\mathrm{kj}}\gamma_{k}^{2}+y_{j}^{1}a^{2}+u_{j}^{2},$$

where:

*y* = costs per IC entitlement per year

1 = granted entitlement

2 = used entitlement

*j* = client *j*

*X*_*i*_ = case‐mix variable i

*M*_*k*_ = dummy for month k

*I* = total number of case‐mix variables

*K* = total number of months in the observation window

$\beta_{0}^{1,2}$, $\beta_{i}^{1,2}$, $\gamma_{k}^{1,2}$, *α*^2^ = parameters for the costs of the IC entitlement used/granted

*u*^1,2^ = stochastic error term for the costs per IC entitlement used/granted.

Practice variation was calculated as shown in Equation (3) (Duell et al., 2017). Practice variation was present if observed minus predicted outcomes were significantly different from zero at a statistical significance level of 0.05. We have used a parametric bootstrap method that has taken account of the sampling variability in the estimated $\hat{y^{1,2}}$ (assuming a normal distribution) to calculate the confidence interval around the average practice variation per region and to obtain appropriate standard errors. Confidence intervals were calculated after bootstrapping 100 times.

(3) $${\mathrm{PV}}_{r}^{1,2}=\sum_{r}\left(y^{1,2}-\hat{y^{1,2}}\right)/N_{r}.$$

Using the same notations as above (after exclusion of the index *j*) and where

${\mathrm{PV}}_{r}^{1,2}$  = Practice variation for care office region *r*,

*y*^1,2^  = Observed value

$\hat{y^{1,2}}$  = Predicted values based on case‐mix variables

*N*_*r*_  = Number of cases in region *r*

On the basis of the above equations, we drew conclusions on the size of the practice variation between care office regions. However, these equations do not provide information on the practice variation within care office regions. In order to learn more about how unequal the practice variation is distributed within these regions, we calculated an inequality index on the basis of the methodology of the Gini Index (Bellù & Liberati, 2005) by (a) calculating the cumulative proportion of the absolute value of practice variation and the corresponding cumulative proportion of the population and (b) computing the inequality index (i.e., by calculating the area that lies between the line of equality and the practice variation curve and dividing this by the total area that lies beneath the line of equality). In turn, we decomposed this inequality index by estimating Equations (1)–(3) while controlling for regions and calculated a new inequality index excluding the region effect. This enabled us to only look at the effect of individual differences in practice variation within care office regions.

#### 3.5.3 Assessing practice variation and ability to pay

In order to explain differences in practice variation between the IC granted and IC used, we investigated whether income and out‐of‐pocket payments were associated with variation in IC use. This was carried out by means of linear regression analyses and computation of practice variation. The analyses can be summarized by means of the following Equations (4‐5) (using the same notations as before):

(4) $$y_{j}^{2}=\beta_{0}^{3}+\sum_{i=1}^{I}X_{\mathrm{ij}}\beta_{i}^{3}+\sum_{k=1}^{K}M_{\mathrm{kj}}\gamma_{k}^{3}+y_{j}^{1}\alpha^{3}+P_{j}\varepsilon^{3}+u_{j}^{3},$$

(5) $${\mathrm{PV}}_{r}^{3}=\sum_{r}\left(y^{2}-\hat{y^{2\mathrm{new}}}\right)/N_{r},$$

where

*P*  = ability to pay (out‐of‐pocket payments and income)

$\beta_{0}^{3}$, $\beta_{i}^{3}$, $\gamma_{k}^{3}$, *α*^3^,*ε*^3^  = parameters for the costs of the IC entitlement used

${\mathrm{PV}}_{r}^{3}$  = practice variation for care office region *r*

*y*^2^  = observed value (costs per IC entitlement used per year)

$\hat{y^{2\mathrm{new}}}$  = predicted values based on equation 4

$u_{j}^{3}$  = stochastic error terms for the costs per IC entitlement.

In addition, the second analysis (Equation 2) regarding the costs per IC entitlement used by client *j* ( $y_{j}^{2}$) was also performed on a dataset stratified by income classes. This was first done without correcting for out‐of‐pocket payments by client *j (* *P*_*j*_) and then correcting for *P*_*j*_. We also calculated inequality indexes to investigate the effect of income and out‐of‐pocket payments on individual differences in practice variation.

### 3.6 Sensitivity analyses

We performed additional analyses in order to investigate the robustness of our results. First, we repeated the main analysis excluding individuals who did not already have a valid entitlement in 2012. This was done in order to exclude a number of individuals who postponed the use of institutional LTC. Second, we replicated the main analysis after we excluded all individuals who did not use any IC in 2013. Third, we performed the main analysis excluding short and low intensity entitlements, which together explain 40% of the entitlements not used. Fourth, we set the personal care budget 2The personal care budget (PCB) is a sum of money which individuals—who need care due to disability, illness, or old age—can use to buy care and hire care providers themselves. Any reimbursement was financed in 2013 from the Exceptional Medical Expenses Act. Only if the Dutch CNAC has established that you are entitled to (institutional) LTC, are you eligible for a PCB. (PCB) as zero use instead of missing observations, while controlling for the fact that those individuals have personal care budgets. Fifth, we replicated our main analysis using a different method, namely by assessing the practice variation in one step, by including dummy variables for each region in Equations (1) and (2). Finally, we re‐estimated our results regarding the ability to pay by analysing its direct association with practice variation. This, to take into account the possible additional effect of income and out‐of‐pocket payments as a mediator between case‐mix factors and LTC use.

## 4 RESULTS

### 4.1 Descriptive statistics

The dataset included 553,819 applications/observations made by 426,934 unique clients, with €38,124.59 on average granted on IC per year per individual in 2013. These clients only used €21,502.91 of IC in the same year, resulting in a gap of €16,621.68 (see Table 1). Most of the clients were female and were 80 to 89 years old (see Table 2 for more information on client characteristics).

**Table 1:** Outcome variables

Variable name	2013
Average cost of IC entitlement granted in 2013	€ 38,124.59
Average cost of IC entitlement used in 2013*	€ 21,502.91

*Note*. 21% of the individuals who were granted IC did not use it (missing observations).

**Table 2:** Client characteristics

Variables	Total
Number of applications	553,819
Number of unique clients	426,934
Age clients by category in years (in %)	
0–9	0.3
10–19	3.7
20–29	8.8
30–39	5.1
40–49	6.3
50–59	7.3
60–69	7.8
70–79	12.6
80–89	30.8
90–99	6.7
≥100	0.6
Marital status (in %)	
married	17.1
unknown	6.6
not married	40.9
widow	34.7
other	0.6
Gender (in %)	
female	58.8
male	41.2
Dominant health problem (in %):	
omatic illness	34.9
psychogeriatric problems	23.7
psychiatric problems	11.5
physical disability	5.4
intellectual disability	23.7
Sensory disabilities	0.8
no dominant health problem registered	0.0
Secondary health problem (in %)	
somatic illness	20.0
psychogeriatric problems	2.0
psychiatric problems	7.8
physical disability	5.4
intellectual disability	1.0
sensory disabilities	1.0
no secondary health problem registered	61.5
psychosocial	0.1
Out‐of‐pocket payment^a^	
average out‐of‐pocket cost per month	€ 100.42
Income classes (in %):	
low (<€15,000 per year)	29.7
under average (€15,000–€30,000 per year)	44.9
average (€30,000–€45,000 per year)	8.0
high (>€45,000 per year)	5.3
unknown/missing	12.6
The type of care package granted deviates from the one used^b^ (in %)	
percentage which deviates	0.1
Personal care budget (%)	
Percentage with a personal care budget	4.4
Non‐institutional long‐term care is granted aside from institutional long‐term care (%)	
Percentage with two forms of care	19.5
IC entitlement valid by month (%)	
Before 2013	63.8
January	66.5
February	68.5
March	70.3
April	71.7
May	73.1
June	73.9
July	75.6
August	77.0
September	78.4
October	79.8
November	81.0
December	81.8

*Note*. Results from a one‐way ANOVA test across care offices show a *p* value smaller than.000 for all variables. The negative income class has been excluded from the analyses due to the fact that the ANOVA did not show a *p* value < .05 and only had 265 observations (=0.0%).

a 11% of the observations were missing.

b 4 % of the observations were missing.

**Graph 1:**
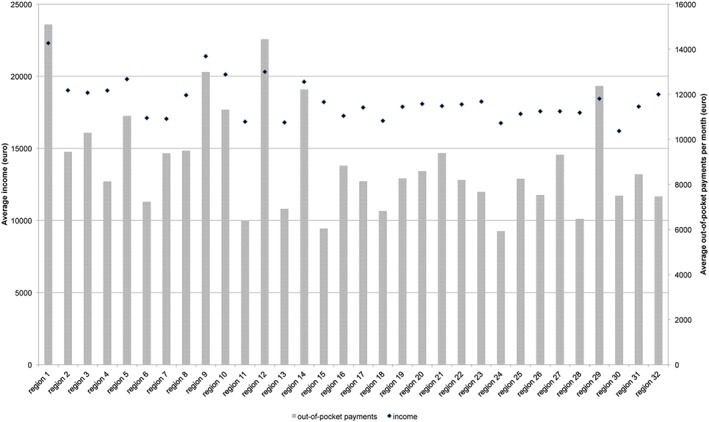
Financial characteristics by care office region [Colour figure can be viewed at http://wileyonlinelibrary.com]

Region 1 had the highest out‐of‐pocket payments and highest average income (see Graph 1). Also, as expected, the highest income group had much higher out‐of‐payments than others, the lowest costs for IC granted and IC used (see Table 3). An ANOVA test across care offices showed a *p* value smaller than.000 for out‐of‐pocket payments and income, indicating that income and out‐of‐pocket payments were not equally distributed across regions.

**Table 3:** Characteristics by income classes

Income class	Number of entitlements	Average out‐of‐pocket payment in euro per month	Average entitlement cost in euro	Average usage cost in euro
Unknown	8,631	240.14	34,097.19	10,964.43
Low	16,4483	12.54	39,916.61	23,359.77
Under average	24,8457	28.45	37,374.03	23,150.18
Average	44,505	225.20	34,507.68	21,908.50
High	29,170	977.85	33,511.74	18,557.59

In 0.3% of the observations, the care office region is unknown.

In addition, we performed stratified regression analyses to estimate the effect of out‐of‐pocket payments on service utilization for the different income groups (see Table 4). All coefficients show that the average out of pocket costs are associated with less use. More specifically, the effect of out‐of‐pocket payments per euro, on use, decreases as the income increases. However, when multiplying the coefficients with the average out‐of‐pocket payments, we can conclude that the highest income class is affected the most by this measure (highest income group: −3.69 × 977.85 versus the lowest income group −106.44 × 12.54).

**Table 4:** Statistical analyses with dependent variable “entitlements used” and the ability to pay of a client as an independent variable

	Income class: low	Income class: below average	Income class: average	Income class: high
	Adjusted *R* ^2^.054	Adjusted *R* ^2^.034	Adjusted *R* ^2^.079	Adjusted *R* ^2^ = .092
	Coefficient	Coefficient	Coefficient	Coefficient
Average out‐of‐pocket cost per month in euro	−106.44*	−30.42*	−12.07*	−3.69*
Constant	24,931.29*	24,188.29*	24,701.89*	22,200.00*

* Statistically significant at a *p* value of <.05.

### 4.2 Practice variation in access and use of LTC across care office regions

**Graph 2:**
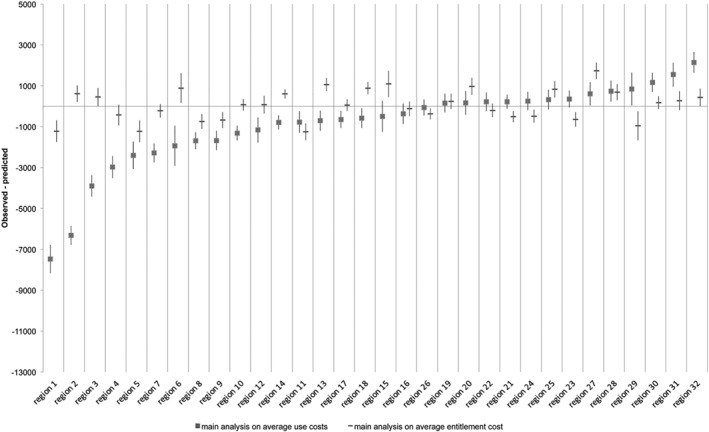
Practice variation in institutional care entitlements granted and used

Graph 2 illustrates that practice variation in IC use was three times larger than in IC granted; practice variation in institutional LTC granted was equal to €2,978.19 and practice variation in institutional LTC used was equal to €9,616.69. This is because the difference between the observed and predicted probabilities for IC use lay more often further from 0 and confidence intervals between regions overlap less. The regression models underlying this graph are shown in Appendix S2. The practice variation in institutional LTC used ranges from −€7,471.64 till €2,145.05, indicating Region 1 uses on average €7,471.64 less than what we would expect. This is 19.6% of €38,124.59, the average cost of the IC entitlement granted in 2013. In addition, it indicates that Region 32 uses on average €2,145.05 more than what we would expect, which is equal to 5.6% of €38,124.59. The practice variation in institutional LTC granted ranges from −€1,252.42 till €1,725.77, indicating Region 11 uses on average €1,252.42 till less than what we would expect, which is equal to 3.3% of €38,124.59. In addition, it indicates that region 27 uses on average €1,725.77 more than what we would expect, which is equal to 4.5% of €38,124.59.

**Graph 3:**
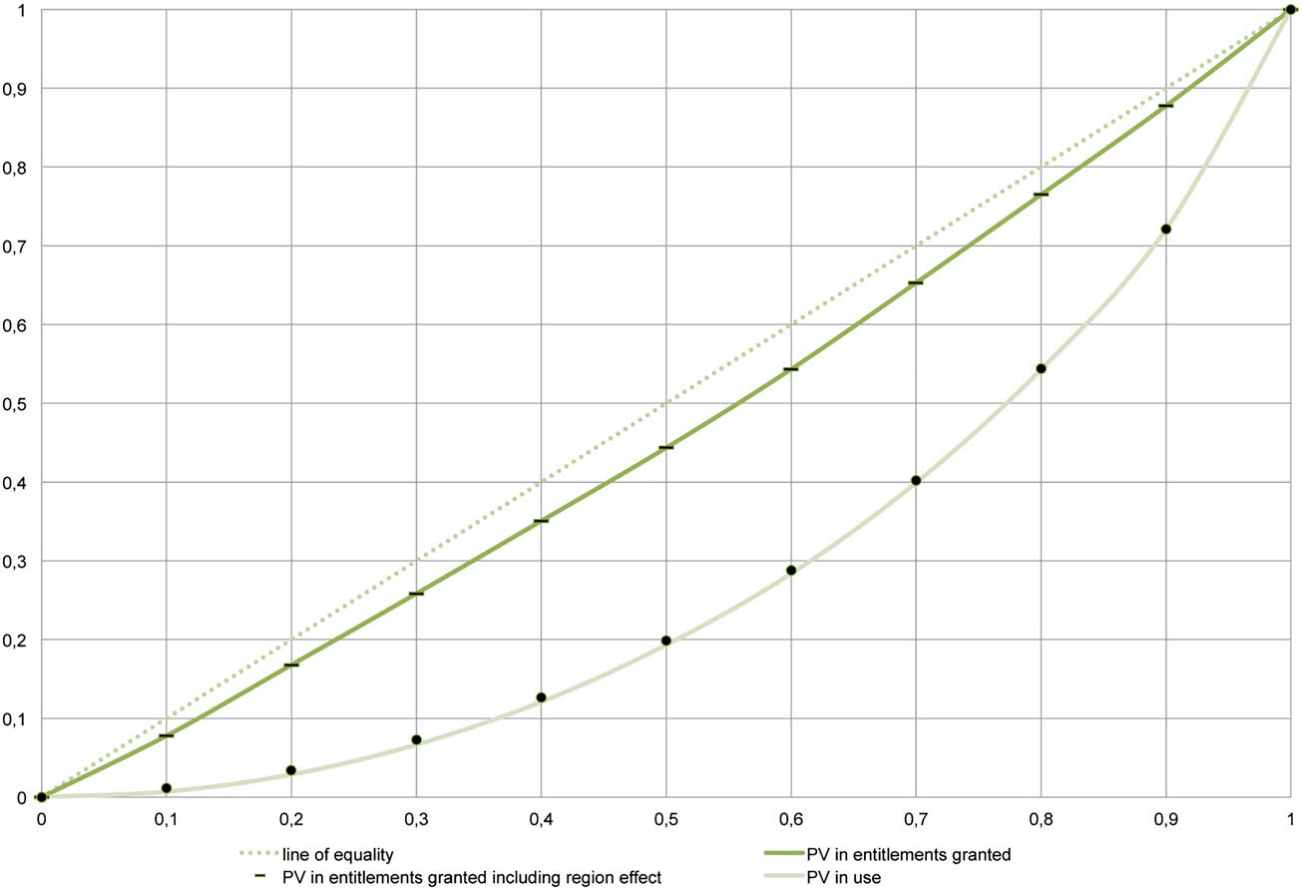
Individual practice variation in institutional care entitlements granted and used [Colour figure can be viewed at http://wileyonlinelibrary.com]

Graph 3 shows that the practice variation in entitlements granted and used was also not equally distributed amongst the individuals. On a scale from 0 (maximal equality) to 1 (maximal inequality) the practice variation we found was 0.072 for the entitlements granted and 0.427 for the entitlements used (from now on: the inequality index). Furthermore, when controlling for a region effect, the inequality index did not significantly change (we found an inequality index of 0.072 for the entitlements granted and 0.420 for the entitlements used). In other words, the inequality in practice variation could not be explained by the variation between care office regions.

### 4.3 Practice variation and ability to pay

**Graph 4:**
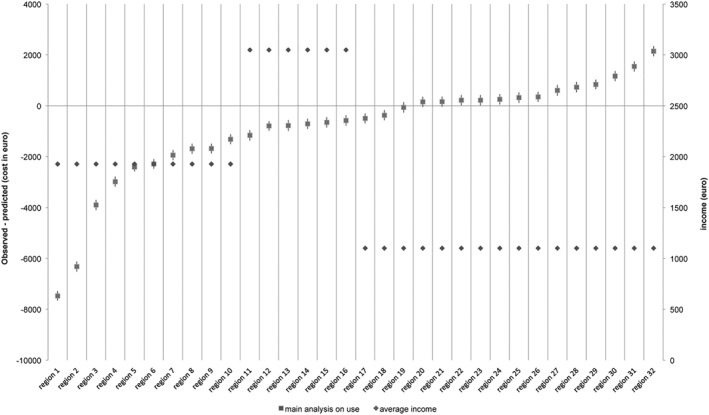
Practice variation in institutional care used relative to income

To investigate how income is associated with practice variation in IC use, we calculated the average income in the following cases: predicted < observed, predicted = observed, predicted > observed (see Graph 4). This graph shows that income was highest in the care office regions without practice variation.

**Graph 5:**
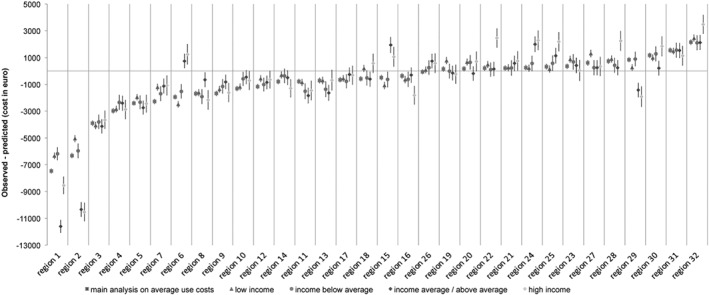
Practice variation in institutional care use stratified by income groups

**Graph 6:**
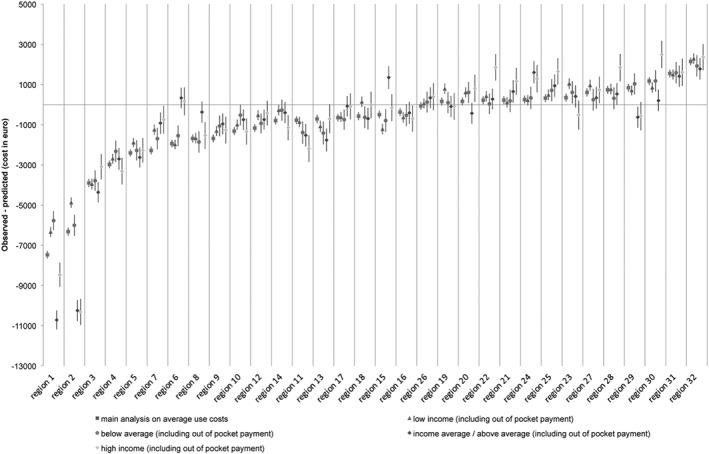
Practice variation in institutional care use stratified by income groups and corrected for out‐of‐pocket payments

Graph 5 shows practice variation in IC use per income group. The shape of the curve does not change much, which indicates that income did not have a large effect on the practice variation between regions. However, income groups did explain variation within some regions. In these cases, income groups had confidence intervals that deviate significantly from each other. Graph 6 shows that including out‐of‐pocket payments, in the stratified analysis, lead to less practice variation in IC use. When taking absolute values of practice variation for (a) low income, the variation decreases by 2%; (b) income below average, the variation decreases by 4%; (c) average income, the variation decreases by 9%; and (d) high income, the variation decreases by 2%.

### 4.4 Sensitivity analyses

Graph 7 (Appendix S3) shows the results of the first sensitivity analysis only including entitlements already valid before 2012. Excluding entitlements granted after 2012 excluded a number of individuals who postponed the use of IC. Graph 8 and 9 (Appendix S3) show the second and third sensitivity analyses excluding all IC granted if there was no use, and excluding short and low intensity entitlements, respectively. Graph 10 (Appendix S3) shows the last sensitivity analysis, including a personal care budget as zero use. The results in all four graphs show that unwarranted variation within a region was influenced in a number of regions, however, the magnitude of unwarranted variation was still about the same. In addition, as discussed in the method session, we performed our main analysis including the regional dummy variables. Plotting the coefficients of the regions exactly yielded the same results as plotted in Graph 2 (excluding the reference region). Finally, the results from the analysis on the direct effect of the ability to pay on practice variation show that the ability to pay has a statistically significant positive effect on the practice variation (Table 5). However, the model has a relatively low adjusted *R* ^2^ of.018. We can conclude that on the basis of these results, the different income groups explain only a small part of the observed practice variation and therefore are not the main determinant of the practice variation in entitlements used between regions. Our main analyses are in line with these findings as they show that income groups are not an important determinant for the overall variation between regions.

**Table 5:** The direct effect of the ability to pay on practice variation

Independent variables	Coefficient Adjusted *R* ^2^ = .018
Income classes	
Low	1,082.00*
Below average	692.60*
High	761.00*
Unknown	−5,808.00*
Out‐of‐pocket payment	
Average out‐of‐pocket cost per month in euro	−5.15*
Constant	674.40*

* Statistically significant at a *p* value of <.05.

## 5 DISCUSSION AND CONCLUSION

In a decade, when the number of people receiving LTC has increased in many countries and in the context of increasing LTC expenses with ageing populations, many countries are investigating how to set up their LTC systems to make them sustainable and keep LTC accessible to those in need. This paper provides (a) more insight into practice variation in granted and used LTC across Dutch care office regions, given that the client has applied for LTC, and (b) more insight into how differences in clients' ability to pay is associated with practice variation in institutional LTC use.

After controlling for differences in need of individuals across care office regions, we conclude that the use of institutional LTC is more unequally distributed between regions as compared with institutional LTC being granted. This result is in line with our first hypothesis and may be explained by the differences in systems for allocating entitlements (a nationally regulated process) and for actually using care (a more locally organized process). The difference in practice variation across Dutch care office regions in 2013 (the observed minus the predicted outcomes) was equal to €2,978.19 for Dutch institutional LTC granted, and equal to €9,616.69 for institutional LTC used. This yielded an overall difference of €6,638.50.

We also found a large amount of non‐usage, about 30%. This high number of non‐usage could partly be explained by non‐usage of short and low‐cost entitlements (about one‐third of the non‐usage). Also, there were a number of entitlements in our dataset without a unique identifier that had to be excluded from the analyses, which could cause an overestimation of the non‐usage effect. Further, we found more institutional LTC being granted than used in combination with more practice variation in use. This could be due to differences across regions in clients not using the institutional LTC granted. An ANOVA test showed that non‐usage is not equally distributed across care office regions. With a sensitivity analysis that excluded short and low intensity entitlements that explain 40% of the non‐usage, we showed practice variation of about the same magnitude as our main analysis and therefore supported our main conclusions. The gap between Dutch institutional LTC need and use was somewhat larger, compared with Ozegowski and Sundmacher's (2014) findings for outpatient care in Germany. To the best of our knowledge, this is one of the few papers that used a so‐called equity index (need divided by use) to calculate the gap. This paper therefore helps to place our findings in context. The equity index of Ozegowski and Sundmacher (2014) ranged from 0.78 to 1.28. Calculating the equity index for our results led to a gap ranging from 1.53 to 2.46, indicating an under‐provision of institutional LTC. However, large numbers of non‐usage are in line with the Dutch literature. Bakx et al. (2016) for instance showed that only 75% of the people eligible for institutional LTC used the granted entitlements, 10% of the Dutch people eligible for institutional LTC do not use it and 15% arranges formal home care, mostly instead of institutional LTC. Both our paper and Bakx et al.'s (2016) paper focus on the gap between need and usage. The gap itself could be the result of the willingness to wait for a preferred provider, or people may apply for an entitlement before they actually intend to use it to avoid being put on a waiting list and/or have one ready in case this is needed in the future (CPB and SCP, 2015). Also, other supply limiting aspects or incidental factors could play a role. Some patients may use care earlier than what we would have expected on the basis of the person's assessed needs and others use it later. And this could depend somewhat on the region where the person lives. To investigate the factors associated with the gap in more depth is an interesting subject that could be investigated in future research.

Furthermore, it remains unclear if all people in need also apply for institutional LTC. If not, our findings may underestimate the true practice variation. For instance, Guthmuller and Wittwer (2017) showed that French people above 30 who are eligible for care are not more likely to visit a doctor in France as compared with non‐eligible people. In the Netherlands, the number of people eligible to receive institutional LTC but who did not apply for care is expected to be low. In the Netherlands, the GP functions as a “gatekeeper”/agent referring a patient to secondary care. Visiting the GP and applying for care at CNAC is free of charge. Also, income‐related inequity plays little to no role in the probability of a GP‐visit. Consequently, people in need of institutional LTC face no meaningful barrier to enter the assessment process. The function of the GP as an agent regarding this process is also supported by data from the CNAC; in 2013, 65% of the applications were initiated by the GP or (out‐patient) LTC providers. Furthermore, only about 5% of applications for IC in the Netherlands were initiated by the patient themselves and even these were likely to be the result of an interaction with a health care provider ([dataset] CNAC, 2014). As a consequence, the application process is accessible for everyone and is not dependent on a clients' income. Therefore, as, (a) the latent demand for LTC driven by the patient seems to be low in the Netherlands and (b) the process of assessing needs and granting entitlements is organized centrally and has a verifiable standardized procedure, we believe that the practice variation found in the paper reflects to a large extent the actual practice variation in institutional care in the Netherlands.

After stratifying by income, we concluded that the practice variation within certain care office regions differed by income group, and controlling for out‐of‐pocket led to less practice variation in institutional LTC use. We found that, in line with our second hypothesis, the higher a clients' income was, the less institutional LTC a client used. It could be the case that poorer individuals use more LTC when differences in needs between poorer and richer individuals are not fully captured. However, we would like to again emphasize that we have access to quite extensive data on needs. Our data covers the most important aspects of needs, as national experts use the same data in support of the needs assessment by CNAC. It could of course be the case that some granularity of the needs data is lost when including this data in our regression analyses. However, we used a very flexible characterization of the data in the regression analyses. Because of that, we still believe the level of detail and scope is as such that the loss of granularity will be negligible and should therefore, to a large extent, reflect the most important aspects of needs.

Another finding regarding the ability to pay is that when decomposing the practice variation by care office regions and stratifying by income group, results did not differ much by income group. In other words, income could explain some of the inequity in access to institutional LTC across regions but was not an important determinant. In addition, we found that, in line with our third hypothesis, income‐related out‐of‐pocket payments somewhat reduced practice variation in institutional LTC across income groups and care office regions.

According to Wennberg, Fisher, and Skinner (2002), there are three types of care: effective, supply sensitive and preference sensitive. Most variation in health care costs across regions usually arises when the effect of the care that is granted is either unknown or marginal (supply sensitive) or heterogeneous (preference sensitive; Skinner, 2011). When applying this typology, the process of using institutional LTC may be classified as sensitive to preferences. This type of care may be provided by other caregivers (informal, private, formal home care), and supply factors seem to play no crucial role in practice variation in the Dutch institutional LTC (Duell et al., 2017). Clients' preferences may be influenced by ability to pay and may therefore explain differences in practice variation between income groups and the decrease in practice variation when taking into account out‐of‐pocket costs. Empirical literature from within the United States, such as Zafar et al. (2013) and Ubel, Abernethy, and Zafar (2013), supports our findings that these out‐of‐pocket costs influence the preferences to use institutional LTC, postpone it or not use it at all. Even though the out‐of‐pocket costs are larger than in the Netherlands, these papers are nonetheless illustrative of the trade‐off a client has to make at a certain level of income. In addition, when specifically looking at the role of out‐of‐pocket cost in the Netherlands, Kasje, Timmer, Boendermaker, and Haaijer‐Ruskamp (2002) showed that preferences of caregivers are also influenced by out‐of‐pocket costs when there was no difference in outcomes, in order to avoid co‐payments for their clients. All of the above led us to conclude, in line with our third hypothesis, that out‐of‐pocket costs have the ability to influence use of institutional LTC, making the LTC system more efficient and more equitable and therefore has a role by decreasing practice variation in the Netherlands. However, it is crucial to monitor the effect of out‐of‐pocket costs at certain income levels in order to avoid possible underuse of high‐value care for those from lower socio‐economic backgrounds.

Furthermore, aside from the sensitivity analysis discussed above, we performed other sensitivity analyses; excluding a number of individuals postponing the use of institutional LTC, excluding the observations with care granted without it being used, and including personal care budget as no use. The results from these additional analyses showed that the practice variation was about the same magnitude as in our main findings. As a result, our main conclusions remain.

To summarize our main conclusions, the Dutch central system of the granting of entitlements showed almost no practice variation, which enabled us to investigate practice variation specifically caused by unequitable use of LTC. Our study demonstrated that local factors such as patient preferences lead to more, but still comparatively limited practice variation. Also, we showed how a co‐payment measure, in line with the theory of diminishing marginal utility, could be used to reduce practice variation across care office regions and income classes, making the LTC system not only more efficient but also equitable. We conclude that practice variation is effectively addressed in the Dutch LTC by separating access from provision and by implementing a national and uniform system for granting access.

## COMPLIANCE WITH ETHICAL STANDARDS

We thank the Dutch Care Needs Assessment Centre (CNAC) for providing us with access to the data used in this study.

## CONFLICT OF INTEREST

The first three authors did not receive any financial grants to conduct this study. The last author had an appointment as a researcher with the CNAC of one day a week. In addition, we would like to emphasize that this study does not necessarily reflect the view of CNAC on unexplained unwarranted variation.

## ETHICS APPROVAL

According to the Dutch law, a study like this does not require approval from an Ethics Review Board, as the study does not fall within the scope of the Medical Research Involving Human Subjects Act (WMO).

## Supporting information


**Appendix S1**. Supporting info item.
**Appendix S2**. Results statistical analyses with dependent variables “entitlements granted” and “entitlements used”.
**Appendix S3**. Supporting info item.
