# Supplementary material for: Practice variation in long‐term care access and use: The role of the ability to pay
Source: Health Econ. 2019 Aug 30;28(11):1277–92. doi: 10.1002/hec.3940 (PMC6852405; doi:10.1002/hec.3940)
Supplement: Supplementary file 1 — Appendix S1. Supporting info item. Appendix S2. Results statistical analyses with dependent variables “entitlements granted” and “entitlements used”. Appendix S3. Supporting info item. [file HEC-28-1277-s001.zip › suup info.docx]

**Appendix 1**

*The entitlements granted are partly are related to the dominant and secondary health problems as these show the care that is needed due to the health problems. Below we explain the different dominant and secondary health problems:*

- Psychogeriatric problems:

Psychogeriatric health problems are a result of a disease or disorder in or of the brain. Often there is an impairment of the emotional capacity, intellectual capacity and / or memory capacity. An example is of a psychogeriatric health problem is dementia.

- Psychiatric problems:

Psychiatric disorders are also called psychological disorders because one or more symptoms of the disorder are caused by factors in the psyche. Examples of psychiatric syndromes are psychosis, schizophrenia and personality disorders. These can lead to mental disorders, for example a mood disorder such as depression.

- Somatic illness:

A somatic disorder or limitation is often caused by a current somatic (physical) illness or condition. If there are permanent limitations, not caused by disorders of the nervous system or the musculoskeletal system (bone / musculature, joints and connective tissue), the health problem somatic illness is applicable.

- Physical disability:

A physical disability is a physical condition, which may or may not be accompanied by the absence of a body part and / or dysfunctional parts of the body. When there are limitations due to disorders of the nervous system and / or the musculoskeletal system (bone / muscular system, joints and connective tissue) where functional improvement is no longer possible (there may still be a deterioration) then the health problem physical disability is applicable.

- Psychosocial:

A psychosocial health problem is a condition with both limitations in social care and impairments in psychosocial functions: memory and thinking, concentration, perception of the environment and motivation.

- Sensory disability:

The health problem sensory disability is applicable when a client has a visual or auditory-communicative disability or a (very) serious speech problem (or disorder). This concerns sensory functions and pain: seeing, hearing, taste, smell and touch, and voice and speech.

- Intellectual disability:

There is a health problem called intellectual disability if 1) the patient scores cognitively / intellectually below average on a general intelligence test (norm: IQ 70 or lower), 2) there are permanent limitations regarding social and life skills and 3) this has arisen before the 18th year of life.

**Appendix 2**

**Table A2. Results statistical analyses with dependent variables “entitlements granted” and “entitlements used”**

| ***Independent variables*** | ***Coefficient if the dependent variable = costs entitlements granted***  ***Adjusted R2 = 0.584*** | ***Coefficient if the dependent variable = costs entitlements used***  ***Adjusted R2 = 0.506*** |
| --- | --- | --- |
| Age clients by category in years: |  |  |
| 0-9 | 10,963* | -3,434* |
| 10-19 | 14,094* | -5,813* |
| 20-29 | 3,702* | -7,483* |
| 30-39 | 5,192* | -6,414* |
| 40-49 | 5,508* | -5,248* |
| 50-59 | 5,787* | -2,662* |
| 60-69 | 5,709* | 174 |
| 70-79 | 3,005* | 1,149* |
| 90-99 | -2,772* | 869* |
| ≥ 100 | -4,176* | 3,151* |
| Marital status |  |  |
| unknown | 2,040* | 1,334* |
| not married | 862* | 1,736* |
| widow | -211 | 752* |
| other | -286 | 304 |
| Gender: |  |  |
| female | 1,813* | 1,247* |
| Dominant health problem: |  |  |
| psychogeriatric problems | 8,961* | 4,186* |
| psychiatric problems | 4,107* | -15,570* |
| physical disability | 8,177* | -252 |
| intellectual disability | 5,040* | -5,466 |
| Sensory disabilities | 13,551* | -3,527* |
| no dominant health problem registered | -18,979* | 792 |
| Secondary health problem: |  |  |
| psychogeriatric problems | 4,888* | 920* |
| psychiatric problems | 5,550* | -560* |
| physical disability | 1,840* | 1,345* |
| intellectual disability | 5,490* | -778* |
| sensory disabilities | 1,790* | -607* |
| psychosocial | -2,582* | -916 |
| no secondary health problem registered | 130* | -102 |
| IC entitlement valid by month |  |  |
| January | 2,574* | 1,449* |
| February | 3,173* | -719* |
| March | 3,307* | -747* |
| April | 3,888* | -719* |
| May | 4000* | -921* |
| June | 4,023* | -465* |
| July | 3,944* | -714* |
| August | 4,150* | -483* |
| September | 4,140* | -561* |
| October | 3,715* | -545* |
| November | 4,699* | -760* |
| December | 5,354* | -173 |
| interaction variables |  |  |
| female * other marital status | 300 | -105 |
| female * unknown marital status | -1,071* | -391 |
| female * not married | -426* | -951* |
| female * widow | 83 | -331* |
| female * age 0-9 | -2,245* | 1,338 |
| female * age 10-19 | -2,315* | 828* |
| female * age 20-29 | -2,695* | 151 |
| female * age 30-39 | -2,088* | 487 |
| female * age 40-49 | -1,477* | 328 |
| female * age 50-59 | -1,374* | -457 |
| female * age 60-69 | -1,273* | -170 |
| female * age 70-79 | -701* | -884* |
| female * age 90-99 | 839* | 425* |
| female * age ≥ 100 | 474 | -498 |
| The care package granted deviates from the care packaged used | 608 | 4,476* |
| long-term care entitlement |  |  |
| costs | - | 1* |
| Formal home care granted | - | -9,087* |
| (Constant) | -5,732* | -2,064* |

** Statistically significant at a p-value of < 0.05*

**Appendix 3**

<Graph 7>

<Graph 8>

<Graph 9>

<Graph 10>
