# Supplementary figures and images for: Practice variation in long‐term care access and use: The role of the ability to pay
Source: Health Econ. 2019 Aug 30;28(11):1277–92. doi: 10.1002/hec.3940 (PMC6852405; doi:10.1002/hec.3940)

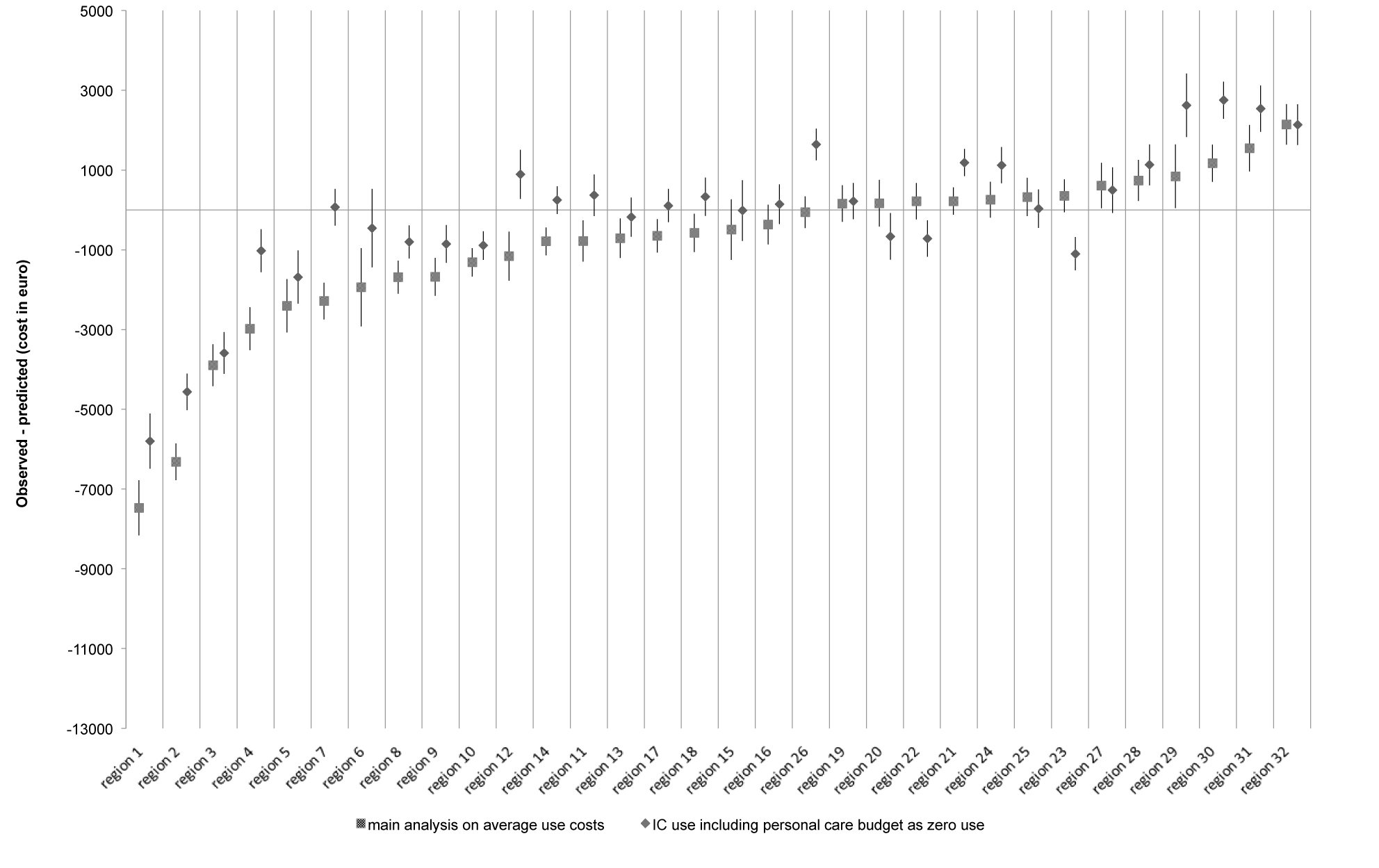

Supplement: Supplementary file 1 — Appendix S1. Supporting info item. Appendix S2. Results statistical analyses with dependent variables “entitlements granted” and “entitlements used”. Appendix S3. Supporting info item. [file HEC-28-1277-s001.zip › Graph_10.tif]

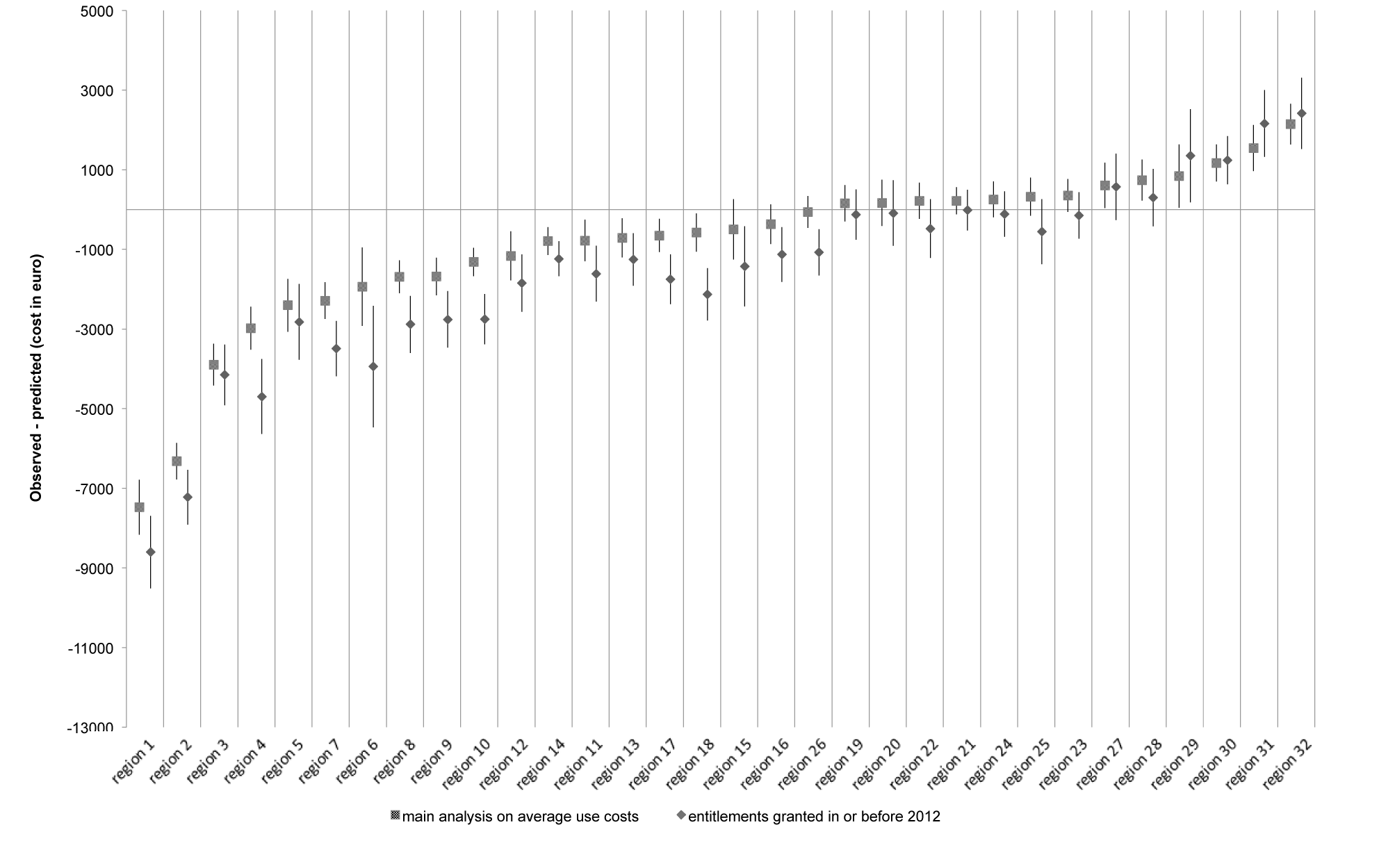

Supplement: Supplementary file 1 — Appendix S1. Supporting info item. Appendix S2. Results statistical analyses with dependent variables “entitlements granted” and “entitlements used”. Appendix S3. Supporting info item. [file HEC-28-1277-s001.zip › Graph_7.tif]

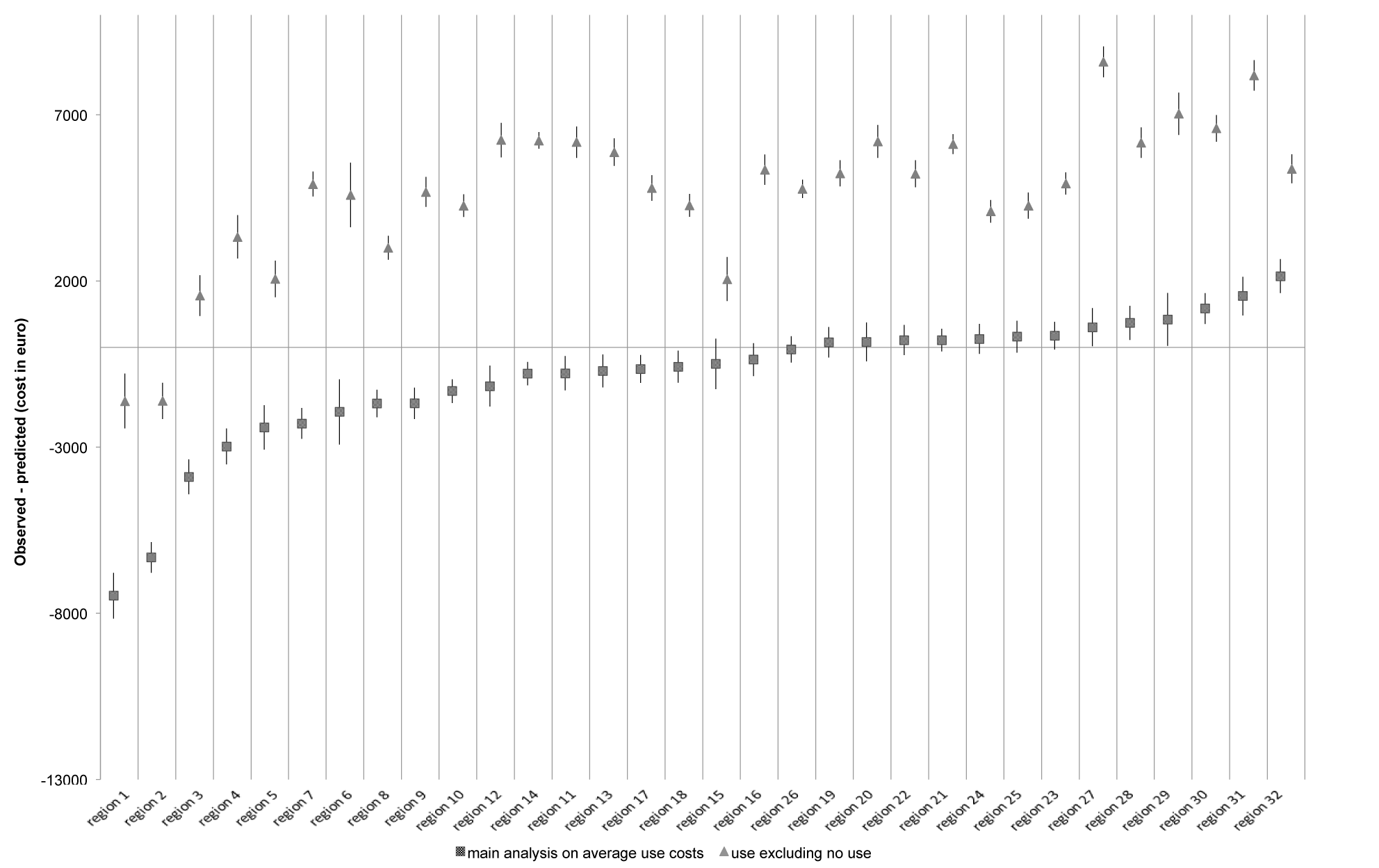

Supplement: Supplementary file 1 — Appendix S1. Supporting info item. Appendix S2. Results statistical analyses with dependent variables “entitlements granted” and “entitlements used”. Appendix S3. Supporting info item. [file HEC-28-1277-s001.zip › Graph_8.tif]

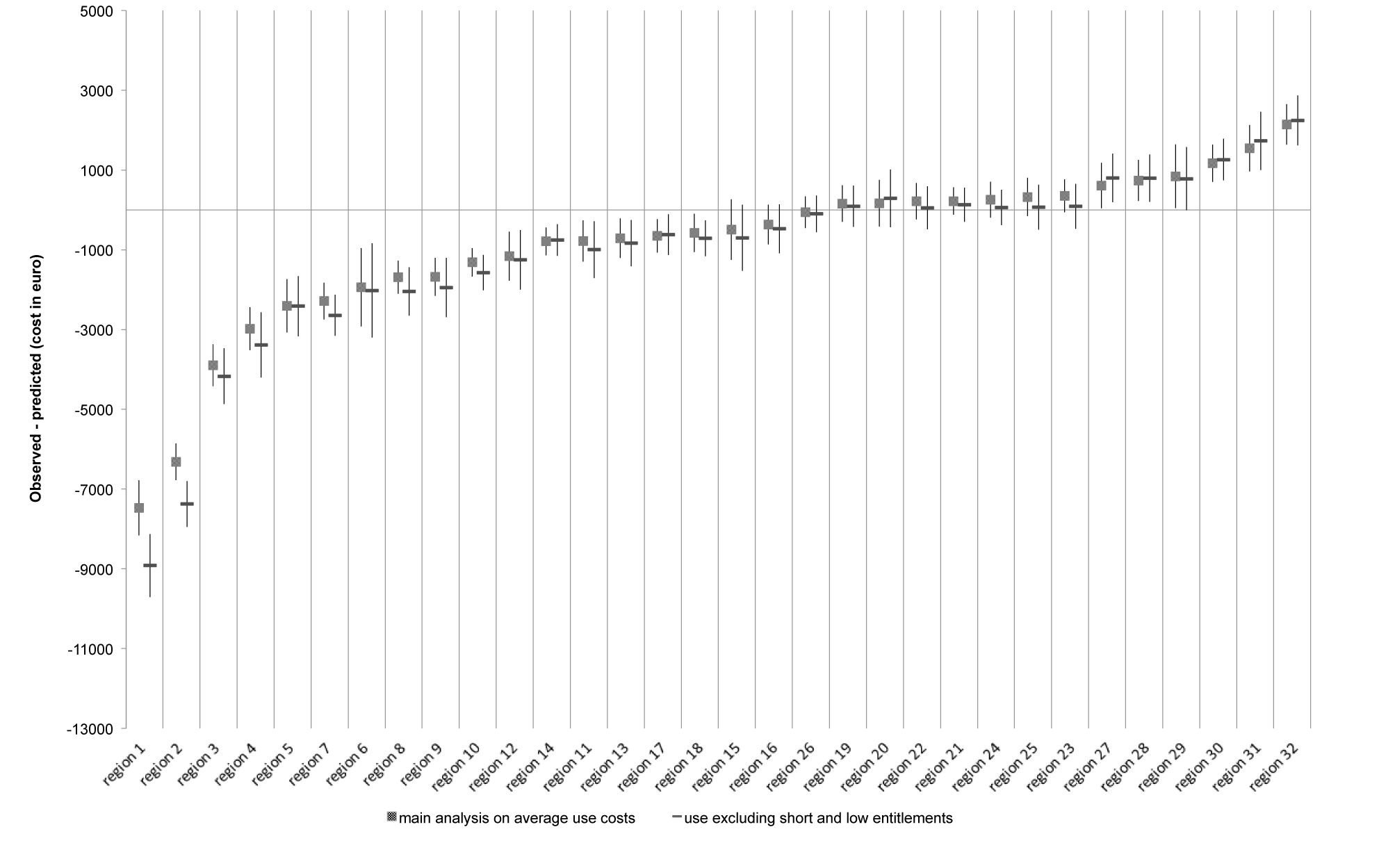

Supplement: Supplementary file 1 — Appendix S1. Supporting info item. Appendix S2. Results statistical analyses with dependent variables “entitlements granted” and “entitlements used”. Appendix S3. Supporting info item. [file HEC-28-1277-s001.zip › Graph_9.tif]
